# Transcriptional regulation of metabolism in disease: From transcription factors to epigenetics

**DOI:** 10.7717/peerj.5062

**Published:** 2018-06-15

**Authors:** Liam J. Hawkins, Rasha Al-attar, Kenneth B. Storey

**Affiliations:** Institute of Biochemistry, Department of Biology, Carleton University, Ottawa, ON, Canada

**Keywords:** Epigenetics, Cancer, Diabetes, Metabolism, Metabolic syndrome, Transcriptional control, Transcription factors

## Abstract

Every cell in an individual has largely the same genomic sequence and yet cells in different tissues can present widely different phenotypes. This variation arises because each cell expresses a specific subset of genomic instructions. Control over which instructions, or genes, are expressed is largely controlled by transcriptional regulatory pathways. Each cell must assimilate a huge amount of environmental input, and thus it is of no surprise that transcription is regulated by many intertwining mechanisms. This large regulatory landscape means there are ample possibilities for problems to arise, which in a medical context means the development of disease states. Metabolism within the cell, and more broadly, affects and is affected by transcriptional regulation. Metabolism can therefore contribute to improper transcriptional programming, or pathogenic metabolism can be the result of transcriptional dysregulation. Here, we discuss the established and emerging mechanisms for controling transcription and how they affect metabolism in the context of pathogenesis. Cis- and trans-regulatory elements, microRNA and epigenetic mechanisms such as DNA and histone methylation, all have input into what genes are transcribed. Each has also been implicated in diseases such as metabolic syndrome, various forms of diabetes, and cancer. In this review, we discuss the current understanding of these areas and highlight some natural models that may inspire future therapeutics.

## Introduction

The identity and function of each cell depends on the expressed genes within its genome. Gene expression plays a major role in mediating virtually every cellular process; for example, cell proliferation, development, aging, responses to external stimuli, and diseases of many kinds. As such, gene expression itself must be under rigorous and coordinated controls to prevent wrongful transcription. Numerous studies have identified multiple mechanisms that are involved in regulating gene expression including the role of the transcriptional machinery, availability of transcription factors, co-factors, and DNA binding elements, accessibility of the DNA to the transcriptional machinery, messenger RNA processing and stability, transportation and translation (Mitchell & Tjian, 1989; Rippe, von Hippel & Langowski, 1995; Guhaniyogi & Brewer, 2001; Butler & Kadonaga, 2002; Villard, 2004; Berger, 2007; Köhler & Hurt, 2007; Bentley, 2014). Abnormalities in any of the above processes may still occur even under stringent regulation which may give rise to various pathologies. Therefore, it is imperative to fully understand the role of transcriptional programming to understand the cause of various medical conditions and find potential remedies. Interest in understanding individual transcriptional mechanisms has been increasing in the past few decades and numerous laboratories are working to understand the effect of various environmental influences, gene mutations, and gene dysregulation in relation to transcription. In this review, we will discuss the effect of transcriptional dysregulation in disease and how understanding transcriptional controls will help solve current medical dilemmas.

## Survey Methodology

In this review, we discuss the intersection of transcriptional control, metabolism, and disease. Our discussion is structured to survey past and current contributions relating to classic transcriptional regulatory mechanisms such as cis- and trans-regulatory elements and the recent emergence of epigenetics with respect to disease metabolism. We add insight into future basic and therapeutic research directions with inspiration from natural systems for metabolic control. We used standard literature search tools (PubMed, Google Scholar, etc.) to identify relevant information and combinatorial keyword patterns to determine research trends.

## Control of Transcription By Cis-Regulatory Elements

There are several regulatory elements within a genome that facilitate transcription. The human genome contains approximately 19,000 protein coding genes (which comprise only ∼1–2% of the entire genome) and a small percentage of non-protein coding genes that serve as regulatory elements (Ezkurdia et al., 2014; Palazzo & Lee, 2015). Gene expression depends on the structure of the genome and whether different genes are accessible to the transcriptional machinery. Studies on epigenetic regulation of the genome have been increasing exponentially in the past decade and emerging work is outlining the role of epigenetics in development, survival, and disease (Jaenisch & Bird, 2003; Esteller, 2007; Portela & Esteller, 2010; Wijenayake & Storey, 2016; Hawkins & Storey, 2018). We will explain the role of epigenetic controls on transcription in detail later in this review.

**Figure 1:**
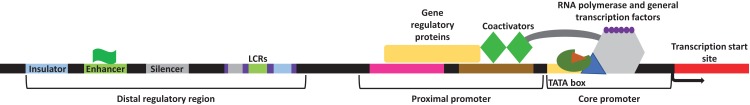
General representation of transcriptional regulatory elements. In eukaryotic transcription, the DNA is present in a three-dimensional orientation where physical interaction between proteins occupying elements of the distal regulatory region and the promoters is possible. Study site: LCRs, locus control regions.

Transcription in eukaryotes is carried out by RNA polymerase II and aided by several general transcription factors that collectively make the transcription initiation complex. Genes usually contain several DNA regulatory elements, called the *cis-regulatory* elements within them. These regulatory sites encompass a promoter region which contains both the proximal promoter elements and the core promoter, and a distal regulatory element region which encompasses the locus control regions (LCRs), insulators, silencers, and enhancers (Maston, Evans & Green, 2006) (Fig. 1). Within the core promoter region is often found the TATA box, a short DNA motif that is within ∼30 nucleotide of the transcription start site in eukaryotes where the general transcription factors will bind. Upstream of the core promoter is the orientation-dependent proximal promoter, where several transcriptional co-activators and regulatory proteins bind to facilitate or prevent transcription. Other *cis-regulatory* elements exist with varying distances to modulate transcription. During development or in response to an external or internal stimulus, some genes are expressed together as a cluster. The LCRs are different *cis-regulatory* elements located within the distal regulatory elements region. These elements function by facilitating the expression of a cluster of genes in a copy-number and tissue-specific manner with respect to the physiological needs of the tissue (Li et al., 2002). Furthermore, LCRs encompass the insulator, silencer, and enhancer elements. Insulators function by “insulating” the expression of specific genes and create a barrier that allows the transcription of the desired gene but blocks the expression of the neighboring gene (Maston, Evans & Green, 2006). Silencers will bind repressors and repress transcription often in an orientation and distance independent manner, although some silencers may be more stringent (Maston, Evans & Green, 2006). Enhancers as their name suggests, will enhance transcription. These *cis-regulatory* elements are located at various distances from the target gene and contain short spans of DNA sequences that function as binding sites for specific transcription factors (both trans-activating and repressing factors) necessary to facilitate sufficient transcription (Sakabe, Savic & Nobrega, 2012). The importance of these *cis-regulatory* elements has been outlined in many cellular processes including embryogenesis, development, and pathological conditions (Villard, 2004; Borok et al., 2010; Sakabe, Savic & Nobrega, 2012; Petit, Sears & Ahituv, 2017). Overall, the presence of these different regulatory elements within the genome creates an intricate network of communications to either facilitate or repress transcription in an organized manner.

As such, while most mutations within the genome are not associated with negative outcomes, a few mutations that occur within the regulatory elements (i.e., the cis/trans-regulatory elements) of specific genes could disturb gene expression, thus resulting in a pathological state. Indeed, genome-wide association studies found that single nucleotide polymorphisms (SNPs) within the enhancer element of different genes is associated with numerous diseases. SNPs within the enhancer element were shown to influence the expression profile of specific genes by altering the transcription factor binding sites for several transcription factors and other regulatory factors associated with transcription (for review, Corradin & Scacheri, 2014). Numerous studies on diabetes, metabolic syndrome, and cancers have shown the involvement of mutations in the cis/trans-regulatory elements as well as microRNAs on the progression of these diseases.

### Cis-regulation of diabetes and metabolic syndrome

Metabolic syndrome is a clustering of pathogenic phenotypes that co-occur in individuals with risk of cardiovascular disease and type 2 diabetes (T2D). In general, definitions include impaired glucose and lipid regulation, increased blood pressure, low HDL cholesterol levels, and increased adiposity (Alberti et al., 2009; Kaur, 2014), and are associated with significantly increased all-cause mortality (Malik et al., 2004; Hui, Liu & Ho, 2010). The prevalence of these conditions is highly dependent on demography, but recent estimates put the number of American adults with metabolic syndrome on an upward trend at almost 35% (Aguilar et al., 2015). Although genetics contribute to some predisposition to the development of metabolic syndrome and each of its components (Pollex & Hegele, 2006; Sladek et al., 2007; Day & Loos, 2011), environmental factors such as a sedentary lifestyle, poor intrauterine conditions, and overnutrition are all highly associative and may turn out to be the best targets for intervention (Aude, Mego & Mehta, 2004; Grundy et al., 2005; Tjønna et al., 2008). As such, while the environment and genetic predisposition can also play a role in disease manifestation, mutations within the non-coding elements of genes can be associated with causing pathologies. One example of such pathology is diabetes. The bone morphogenic protein receptor 2 (BMPR2) is a member of the transforming growth factor beta (TGFβ) receptor family that has been found to play a significant role in pulmonary arterial hypertension (PAH), obesity, and insulin resistance (Schleinitz et al., 2011; West et al., 2013, 2014; Viales et al., 2015). Indeed, studies have found that over 80% of heritable PAH and 20% of idiopathic PAH patients have mutations within their *BMPR2* gene (Machado et al., 2009; Pfarr et al., 2011). A recent study on families with history of PAH showed mutations within the *BMPR2* gene as well as a novel mutation within the promoter of *BMPR2* (c.669G>A) (Viales et al., 2015). The same study concluded that patients that carried both mutations had heritable PAH and manifested severe clinical signs of this disease. Moreover, distal regulator elements were shown to play a significant role in regulating gene expression with respect to obesity. For example, SNPs within long-range enhancers in the fat mass and obesity-associated protein (FTO) directly affected the expression of *IRX3* (Smemo et al., 2014). IRX3 is a transcription factor that has been previously shown to modulate body mass and composition, as shown by the lean phenotype of IRX3 deficient mice (Smemo et al., 2014). The same study showed that mice deficient in IRX3 have an increase in their basal metabolic rates and are prone to fat mass loss.

Pancreatic dysregulation is associated with various forms of diabetes and is partly mediated by abnormal transcription (Federici et al., 2001; Weedon et al., 2014; Stitzel et al., 2015). The pancreas is composed of two major compartments: the exocrine and endocrine compartments. The exocrine compartment is divided into two major cell types; the acinar cells which secrete digestive zymogens and the epithelial cells which secrete bicarbonate in order to neutralize the stomach acids entering the small intestine (Bastidas-Ponce et al., 2017). The endocrine compartment of the pancreas is responsible for secreting appropriate hormones to regulate glucose metabolism and homeostasis. The islets of Langerhans are composed of several cell types which mainly include the alpha, beta, delta, pancreatic polypeptide, and epsilon cells and carry out the endocrine function of the pancreas (Stitzel et al., 2015; Bastidas-Ponce et al., 2017). These endocrine cells mediate glucose homeostasis by secreting the hormones glucagon, insulin, somatostatin, pancreatic peptide or ghrelin (Bastidas-Ponce et al., 2017). While the coordinated secretion of these hormones regulates metabolic homeostasis in organisms, improper or imbalance in secretion of any of these hormones can promote responses characteristic of diabetes mellitus. Adequate and appropriate secretion of these hormones relies on the proper development of the associated cells, therefore any mutations that halt or interrupt the development of these cells can lead to diabetic pathologies. For example, the pancreas-specific transcription factor 1a (PTF1a) is responsible for proper formation of the pancreas during embryonic development and is crucial for the development of both the endocrine and the exocrine compartments (Masui et al., 2008; Weedon et al., 2014; Gonc et al., 2015). Using linkage analysis and whole-genome sequencing of patients with pancreatic agenesis, it was shown that several recessive mutations within the developmental enhancer region of the *PTF1A* gene abolishes transcription factor binding, thus leading to improper development of the pancreas, causing diabetes (Weedon et al., 2014). Moreover, the *BLK* gene encodes for a nonreceptor tyrosine-kinase belonging to the Src family of proto-oncogenes (Gauld & Cambier, 2004). This gene was also found to be expressed in various cell types including beta cells where it promotes insulin synthesis in response to glucose uptake (Borowiec et al., 2009). Patients with maturity-onset diabetes of the young (MODY) were shown to have noncoding mutations within the *BLK* locus which resulted in disruption in insulin synthesis when glucose levels were high (Borowiec et al., 2009). The same study showed that overexpression of *BLK* in MIN6-beta cells caused an increase in the protein levels of two transcription factors, PDX-1 and NKX6.1. PDX-1 was previously shown to regulate the expression of several beta cell genes including *NKX6.1* (Wang et al., 2001). NKX6.1 regulates glucose-simulated insulin secretion in beta cells and together with PDX-1 enhance mass as well as the function of the pancreatic beta cells (Wang et al., 2001; Schisler et al., 2005).

### Cis-regulatory elements in cancer

As medical science advances and screening for genetic abnormalities becomes easier, more and more information is being accumulated on the origins of various diseases and syndromes and how normal metabolic processes are affected under such circumstances. The completion of the encyclopedia of DNA elements (ENCODE) helped with providing a more thorough understanding of various regulatory elements within the human genome (ENCODE Project Corsortium, 2012; Roadmap Epigenomics Consortium et al., 2015). Data repositories like this provided the much-needed step forward in comparing normal vs. “abnormal” metabolic regulation that occur within a cell’s genome. Recently, several studies have been published that focus on the effect of mutations within the *cis-regulatory* elements and how these mutations affect transcription (Babitt et al., 2006; Horn et al., 2013; Perera et al., 2016). For example, whole-genome sequencing of several malignant melanomas showed that in 17 of 19 cases analyzed mutations (C228T and C250T) were found within the telomerase reverse transcriptase (*TERT*) promoter (Babitt et al., 2006; Berger, 2007). Another independent study on the *TERT* promoter of melanomas showed that 125 of the 168 human cancer cell lines screened contain the same somatic mutations (Horn et al., 2013). These mutations generated a *de novo* consensus sequence within 100 base pairs of the transcriptional start site that binds to the E-twenty-six transcription factors within the promoter of the *TERT* gene (Babitt et al., 2006; Horn et al., 2013). Under normal circumstances telomerase activity is repressed in most human cells; therefore, chromosomes are shortened with every round of cell division and this leads to either halting replication or promoting apoptosis when telomeres become shorter in length (Shay & Wright, 2011; Chen et al., 2018). When cells are cancerous, their telomerase activity is significantly increased which allows the chromosomes to maintain lengthy telomeres, thereby immortalizing the cells (Hahn & Meyerson, 2001; Shay & Wright, 2011). Moreover, compared to the wild-type *TERT* promoter, the mutations observed increased the transcription of this gene by 2 to 4 fold (Babitt et al., 2006). Altogether, these studies show that mutations within the promoter region of the *TERT* gene allow an overall increase in production and therefore activity of telomerase, which accounts for yet another explanation of how cancer cells maintain lengthy telomeres (Liu, Yuan & Xu, 2016). The human TERT is also involved in regulating the pentose phosphate pathway; where NADPH and other co-factors required for fatty acid synthesis, RNA synthesis and lipogenesis are generated (Ahmad et al., 2016). The same study showed that inhibition of TERT attenuated the phosphorylation of glycogen synthase in addition to attenuating the expression of glucose-6-phosphate dehydrogenase and transketolase in glioma cells. These findings were congruent with the overexpression of transketolase and decreases in glycogen levels in glioblastoma multiforme patients harboring the C228T and C250T mutation in their TERT promoter (Ahmad et al., 2016). As such, the presence of such mutation does not only promote cancer cell mortality by increasing telomerase activity but also by reprograming the cell’s metabolic response to promote its progression.

## Control of Transcription by *Trans-Regulatory* Elements

*Trans-regulatory* elements are other factors that contribute to regulating transcription under a variety of conditions. While mutations within the *cis-regulatory* elements alone can be associated with diseases, mutations within the proteins that bind those elements can also be associated with different pathologies. TFIID is part of a multi-subunit complex that contains a TATA-binding protein along with other factors that assemble at the promoter of genes to recruit RNA polymerase II to the transcription start site. Subsequent transcription only requires RNA polymerase II, TFIIF and TFIIB as TFIID, TFIIH and Mediator are retained within the promoter. While this assembly is sufficient for basal levels of transcription, activators can bind to enhancers upstream of the promoter and increase the expression of their target genes (Ma, 2011). These activators are also called transcription factors and they bind either as monomers, heterodimers or homodimers to a 6–12 nucleotide consensus sequence within the enhancer region upstream of the core promoter to enhance the transcriptional activity of target genes. Other studies have also shown that activators have the ability to recruit chromatin modifiers in order to facilitate transcription (Bowman & McKnight, 2017). While mutations within the binding sites of transcription factors can prevent protein-DNA complex formation; mutations within the transcription factors themselves are also associated with disease development. Co-activators are intermediary proteins or complexes that can interact with activators and enhance transcription (Kim et al., 2001b; Li & Susztak, 2018). Some coactivators have been shown to increase transcription by promoting the recruitment of RNA polymerase II or activators whereas others recruit chromatin modifiers that relax the structure of the DNA for the transcriptional machinery (Krasnov et al., 2016). Alternatively, repressors and corepressors also play roles in regulating transcription. Repressors inhibit gene expression selectively by binding to the repressor element of the DNA thus preventing the binding of the transcriptional machinery or by recruiting enzyme modifiers that would render the DNA inaccessible to the transcriptional machinery. Transcription partly depends on the interplay between activators and repressors, therefore any inappropriate association of these regulators with their DNA binding elements can result in unfavorable consequences.

### Trans-regulatory elements in diabetes and metabolic syndrome

Maturity-onset diabetes of the young (MODY) is a rare monogenic disorder that follows an autosomal-pattern of inheritance and occurs in individuals younger than 25 years of age (Amed & Oram, 2016). The onset of MODY is not dependent on insulin but is caused by mutations in several genes that result in defective beta cells. As such, one of the major clinical features of MODY is hyperglycemia; however, since MODY shares common features as T1D and T2D, it is often misdiagnosed (Amed & Oram, 2016). The most common mutations associated with MODY occur in at least one of the following components: HNF4α (hepatocyte nuclear factor 4 alpha, MODY1), glucokinase (MODY2), HNF1α (MODY3), insulin promoter factor 1 (IPF-1, MODY4), HNF1β (MODY5) or neurogenic differentiation 1/β-cell E-box transactivator 2 (NeuroD1, BETA2, MODY6) (Fajans, Bell & Polonsky, 2001). HNF proteins are transcription factors that are enriched in the liver but can be also found in several other tissues such as kidney and pancreatic islets (Fajans, Bell & Polonsky, 2001). These transcription factors are thought to play a significant role during development and adulthood. In beta cells, the insulin gene and other genes involved in the transport of glucose and metabolism are under HNF control. In the liver, these transcription factors regulate lipoprotein biosynthesis and fatty acid metabolism (Stoffel & Duncan, 1997; Dukes et al., 1998; Miura et al., 2006; Martinez-Jimenez et al., 2010; Yin et al., 2011). A previous study showed that 50% of Norwegian families with mutations in their HNF1α have clinical MODY3 (Bjørkhaug et al., 2003). The same study showed that two of these mutations prevented the transcription factor from binding to DNA and five mutations prevented the transcription factor nuclear translocation thereby reducing transcription of the target genes in both cases. Moreover, mutations in the glucokinase enzyme are also associated with MODY. Glucokinase is a glucose sensing enzyme that functions to transform glucose from the blood to glucose-6-phosphate in beta cells (Amed & Oram, 2016). As such, inactivating mutations within the glucokinase gene typically manifest hyperglycemia (Froguel et al., 1993). Moreover, the IPF-1 transcription factor has been shown to regulate the transcription of several targets including the insulin, glucokinase, and the glucose transporter 2 genes (St-Onge, Wehr & Grusst, 1999). For this reason, mutations within the IPF-1 gene that abolish its transcriptional activity could result in the onset of various diabetic pathologies including MODY. A case study showed that autosomal dominant mutations within the NeuroD1 (BETA2) gene were causing a dysregulation in the expression of the insulin gene, leading to the development of diabetes before the age of 25 (Malecki et al., 1999). In another case, dysregulation in PGC-1α, a coactivator of *PPARγ* and *PPARα* which are involved in adipogenesis and mitochondrial fatty acid oxidation respectively, was shown to increase the risk of type II diabetes (Ek et al., 2001). Furthermore, PGC-1α was shown to play a role in improving glucose homeostasis and fuel storage in skeletal muscle (Wende et al., 2007; Summermatter et al., 2013). PGC-1α can bind to the MEF2C transcription factor and function as a co-activator where it will facilitate the expression of *GLUT4* (glucose transporter 4) (Michael et al., 2001). The polymorphism Gly482Ser decreases the binding affinity of PGC-1α to MEF2C, thereby decreasing *GLUT4* expression and increasing the risk of developing type II diabetes (Hara et al., 2002; Muller et al., 2003; Zhang et al., 2010).

Differential activities in certain transcriptional repressors can also be associated with metabolic abnormalities. For example, FOXA2 (Forkhead box A2, also known as HNF3β) is a known transcription factor that plays a role in regulating insulin secretion, sensitivity and glucose/lipid metabolism (Lantz et al., 2004; Puigserver & Rodgers, 2006). Recently, several studies have identified FOXA2 as a transcriptional repressor where its association with specific genes prevents their transcription (Rausa, Tan & Costa, 2003; Guo et al., 2012). For example, *FTO* is an important gene that has been associated with regulating body mass. A study by Guo and colleagues showed that *FOXA2* overexpression was associated with significant downregulation of *FTO* promoter activity whereas FOXA2 depletion allowed *FTO* expression, thereby providing evidence that FOXA2 is a negative repressor of the *FTO* gene (Guo et al., 2012). *FTO* was previously shown to be involved in several metabolic syndrome-related conditions such as diabetes, insulin signaling, lipogenesis, and mitochondrial dysfunction, whereby *FTO* overexpression was observed (Bravard et al., 2011; Akbari et al., 2018). In this case, the lack of appropriate repressor response was associated with untimely expression of target genes, which resulted in clinical manifestation of diseases.

### Trans-regulatory elements in cancer

Numerous studies have outlined the role of *trans-regulatory* elements in cancer development (Girnun, 2012; Bradner, Hnisz & Young, 2017; Sciortino et al., 2017; Safe et al., 2018). The availability of tools to easily sequence cancer genomes has provided ample information on the mutations and dysregulations that promote cancer development. Transcription factors play a significant role in differentiation and determining cell fate. For example, P53 is a transcription factor that is responsible for expressing target genes that inhibit the cell cycle and induce apoptosis; however, studies have shown that this transcription factor is one of the most commonly mutated in cancers (Sionov & Haupt, 1999; Ozaki & Nakagawara, 2011; Vogiatzi et al., 2016). Under normal circumstances, P53 is present at low levels, but upon induction of DNA damage, its expression and activity increase (Sionov & Haupt, 1999; Ozaki & Nakagawara, 2011; Vogiatzi et al., 2016). The cell cycle arrest properties of P53 helps to repair DNA damage under circumstances that promote genomic instability and the pro-apoptotic properties of this transcription factor work to eliminate those cells that carry a substantial amount of DNA damage in order to prevent the transfer of deleterious mutations to daughter cells (Ozaki & Nakagawara, 2011). Several mutations within P53 abolish its tumor suppressing activities and promote its tumor progressive function (Gasco, Shami & Crook, 2002; Lim et al., 2009; Ozaki & Nakagawara, 2011; Vogiatzi et al., 2016). Indeed, over 50% of cancers carry mutations within the *P53* gene, however these mutations in most cancers create a stably functional P53 that functions better than the wildtype P53, is resistant to drug treatment, and can cause metastasis (Brosh & Rotter, 2009; Oren & Rotter, 2010). The role of P53 has been studied extensively and one study showed that cancerous P53 was associated with gain of function mutations whereby it caused the expression of ectonucleoside triphosphate diphosphohydrolase 5 (*ENTPD5*) (Vogiatzi et al., 2016). The same study found that ENTPD5 function promotes the folding of N-glycoproteins which is necessary for the proliferation, tissue remodeling, and metastasis of tumors. Furthermore, P53 also plays a role in metabolism by inducing the expression of metabolic genes and suppressing the production of reactive oxygen species (Li et al., 2012). Interestingly, several studies have demonstrated that while some mutations may interrupt the activity of P53 in regulating the cell cycle and apoptosis, the same mutations may not necessarily have a negative influence on its activity in regulating the uptake and catabolism of glucose and suppressing the production of reactive oxygen species (Li et al., 2012; Napoli & Flores, 2017; Itahana & Itahana, 2018). As such, mutations within the P53 gene may differentially regulate its transcriptional activity and favor the expression of a subset of genes leading to metabolic deregulation necessary for oncogenesis (Gatto, Schulze & Nielsen, 2016).

Moreover, HBP1 is a transcriptional repressor that regulates cellular proliferation and inhibition of the Wnt signaling pathway (Shih, Tevosian & Yee, 1998; Sampson et al., 2001; Shih et al., 2001; Escamilla-Powers et al., 2010). Repressors also play a significant role in controlling transcription. HBP1 binding to the promoters of *WNT*, *β-catenin*, *GSK3β* or *LEF/TCF* prevents their expression, thereby rendering Wnt signaling ineffective (Sampson et al., 2001). In addition, the same study showed that HBP1-mediated Wnt signaling suppression prevented cyclin D1 and c-myc gene expression. A decrease in HBP1 levels has been associated with enhanced invasion and tumorigenesis in breast cancers, partly by causing a dysregulation in Wnt signaling (Paulson et al., 2007). Moreover, HBP1 was also reported to play a role in regulating mitochondrial biogenesis and regulation. Indeed, cells deficient in *Hbp1* showed a significant decrease in mitochondrial respiration and an approximate 29% increase in the oxygen consumption rate (Dong et al., 2016). As such, mutations within HBP1, that could interrupt its activity could result in serious cellular damage.

## Microrna Regulation of Transcription

The human genome contains both coding and non-coding RNAs. Apart from transfer and ribosomal RNAs, multiple types of regulatory non-coding RNAs exist (Kowalczyk, Higgs & Gingeras, 2012) including long non-coding RNAs and microRNAs. These two types of non-coding RNAs have been shown to play a significant role in maintaining cellular homeostasis and dysregulation of their activity has been linked to different disease conditions (Li et al., 2010; Ardekani & Naeini, 2010; Esteller, 2011; Wang, 2013; Huarte, 2015; Sun & Wong, 2016; Feng, Xing & Xie, 2016). For the purposes of this review, we will focus on the role of microRNAs in regulating metabolism and disease.

MicroRNAs are small non-coding RNAs that negatively regulate gene transcripts (He & Hannon, 2004) and have regulatory actions in huge numbers of cell functions including development, cellular metabolism, stress response, and disease (He & Hannon, 2004; Wienholds & Plasterk, 2005; Ardekani & Naeini, 2010). MicroRNAs are originally transcribed as pri-microRNAs that are then cleaved by the Drosha enzyme to form a 70 nucleotide long precursor microRNA containing hairpin structures (He & Hannon, 2004). These precursors are then transported to the cytoplasm via exportin 5 where they are cleaved further to form short (21–25 nt) single stranded RNAs that are capable of binding to the RNA-induced silencing complex and target mRNA transcripts based on complementarity (He & Hannon, 2004). The fate of the mRNA transcript is determined by the degree of complementarity between the microRNA and its target transcript. If complementarity between the two is high, the microRNA will bind to the 3′UTR of the transcript and direct it to be degraded, whereas if there is partial complementary, the transcript will be sequestered in p-bodies and stress granules for future retrieval (Liu et al., 2005). Studies have shown the involvement of microRNAs in several cellular processes including autophagy, apoptosis, diabetes, cancer, muscle atrophy, and development (Li et al., 2010; Ardekani & Naeini, 2010; Wang, 2013; Su et al., 2015; Feng, Xing & Xie, 2016). Each microRNA can have several different mRNA targets whereas each mRNA can be targeted by multiple microRNAs. MicroRNAs can target the transcripts of several transcription factors and other *cis-regulatory* elements, thereby preventing them from regulating transcription appropriately. Moreover, microRNAs can also inhibit the mRNA transcripts that are produced by certain transcription factors with respect to the cell’s environment. While regulation by microRNA can be crucial for survival and development, dysregulation of this regulatory factor can be associated with disease.

### MicroRNA in diabetes and metabolic syndrome

With the advancement of technology and the availability of several cancer genomes, it is becoming easier to decipher the role of non-coding RNAs in cells. Pancreatic beta cells play a significant role in maintaining glucose homeostasis by releasing insulin and several microRNAs have been shown to promote apoptosis in beta cells, thereby leading to diabetes (Feng, Xing & Xie, 2016). The programmed cell death protein 4 (PDCD4) is involved in both translation (by suppressing translation initiation) and transcription (by binding to promoters) (Ozpolat et al., 2007; Lankat-Buttgereit & Göke, 2009; Jo et al., 2016). A study by Ruan et al. (2011) showed that nuclear factor κB (NFκB) promotes the expression of miR-21, where this microRNA will then prevent the expression of PDCD4. PDCD4 deficiency in islet beta cells was associated with a decrease in proapoptotic *BAD*, *BAX*, and *BID* transcript levels while increasing the mRNA expression of the antiapoptotic *BCLXL* (Ruan et al., 2011). This example shows how transcriptional regulation of miR-21 by NFκB indirectly prevented apoptosis and abolished pancreatic beta cell apoptosis to prevent development of type 1 diabetes. Moreover, miR-30d was shown to be regulated by glucose and is involved in promoting insulin gene transcription (Tang et al., 2009). A follow up-study showed that miR-30d caused an increase in MAFA, a key transcription factor that increases the expression of the insulin gene in beta cells (Zhang et al., 2005; Zhao et al., 2012). It was also reported that the tumor necrosis factor α (TNF-α) suppresses insulin transcription (Tsiotra, Tsigos & Raptis, 2001; Lawrence et al., 2011). Interestingly, Zhao et al. (2012) found an increase in MAFA when miR-30d levels were high since this microRNA targets a member of the TNF-α activated kinase, the mitogen-activated protein 4 kinase 4 (MAP4K4), thereby alleviating an inhibitory effect on MAFA. This is yet another regulatory mechanism whereby the expression of one microRNA positively regulates transcription by targeting the specific inhibitory factors involved.

Obesity is associated with inflammatory and non-inflammatory factors that are regulated by various mechanisms. Gene expression of different targets associated with obesity is regulated at various molecular levels and is induced with respect to environmental stimuli. The C–C motif chemokine ligand 2/monocyte chemoattractant protein 1 (CCL2/MCP-1) has been documented to regulate metabolism, inflammatory responses, obesity, and insulin resistance amongst others (Rull et al., 2010). A study by Arner et al. (2012) showed several microRNAs that had differential regulation in obesity, nine of which were shown to affect CCL2 secretion from adipocytes. The same study found that overexpression of several microRNAs caused a reduction in CCL2 secretion. MiR-1126 regulated CCL2 expression by associating with its 3′ untranslated region while miR-193b also regulated CCL2 expression by targeting other transcription factors associated with inducing its expression (Arner et al., 2012). This is yet another example of how microRNAs can both directly and indirectly affect the expression of target genes by modulating the activity of other regulatory elements that facilitate transcription.

### MicroRNA and cancer

A recent study showed that resistance to chemotherapeutic drugs via P53 may be mediated by microRNAs (Donzelli et al., 2012). The study found that mutant P53 is responsible for inducing the expression of miR-128-2 that can, in turn, lead to inhibition of apoptosis and thereby promote resistance to several cancer therapeutics such as cisplatin, doxorubicin, and 5-fluorouracyl treatments. For example, miR-128-2 targets the transcript of *E2F5*, a transcriptional repressor to *p21^waf1^* (Donzelli et al., 2012). The same study showed that miR-128-2 binds to the *E2F5* transcript, thereby preventing it from being translated into proteins. A decrease in *E2F5* protein levels was associated with the induction of the *p21^waf1^* promoter activity, leading to overexpression of this anti-apoptotic target in response to the therapeutics. Furthermore, other studies have shown that inappropriate downregulation of specific microRNAs can cause an increase in the expression of their targets; thereby causing a systemic imbalance. For example, miR-101 has been shown to regulate the expression of *SUB1/PC4* (Chakravarthi et al., 2016). SUB1/PC4 has several functions including DNA replication, repair and transcription but for the purposes of this review, we will restrict our discussion to its transcriptional co-activating functions (Conesa & Acker, 2010). As a co-activator, SUB1/PC4 regulates transcription by facilitating the interaction between the general transcription machinery and activators, however the role of SUB1/PC4 in promoting transcriptional repression is also documented (Werten et al., 1998; Conesa & Acker, 2010). A study showed that overexpression of SUB1 in prostate cancer was associated with cellular proliferation by facilitating the expression of several oncogenes (Chakravarthi et al., 2016). The same study reported that a decrease in expression of miR-101 was responsible for the increase in SUB1/PC4 levels; thereby elucidating a control mechanism whereby the wrongful overexpression of a coactivator can manifest in cancer progression. Moreover, a study by Majid et al. (2010) outlined a novel role for microRNAs in tumor suppression. This study showed that overexpression of miR-205 was associated with induction of apoptosis, proliferation arrest, and impairment of metastatic abilities. More interestingly, miR-205 was shown to associate with the promoters of *IL24* and *IL32*, thereby inducing their expression. Both IL24 and IL32 have tumor suppressing properties where reduction in IL24 levels has been observed in breast cancers and was associated with poor prognosis and severe clinical outcomes (Patani et al., 2010). IL32 showed conflicting data as some studies have showed its involvement in inducing T cell apoptosis while others show its activation to be linked to metastasis in colorectal cancers (Goda et al., 2006; Yang et al., 2015). Regardless of its pro- or anti-cancer functions, IL32 expression was shown to be enhanced by miR-205 expression as this microRNA was shown to bind to the *IL32* promoter (Majid et al., 2010).

Hence, transcription can be partly regulated by *cis* and *trans-regulatory* elements as well as microRNAs. The proper communication between these factors is necessary for appropriate gene expression and any dysregulation in one or more of these factors could manifested in a pathological condition. Regulation of transcription happens at many levels and while we discussed the role of *cis*/*trans-regulatory* elements as well as microRNAs, it is also imperative to cover the role of epigenetic regulation in mediating transcription.

## Epigenetic Regulation of Metabolism in Disease

**Figure 2:**
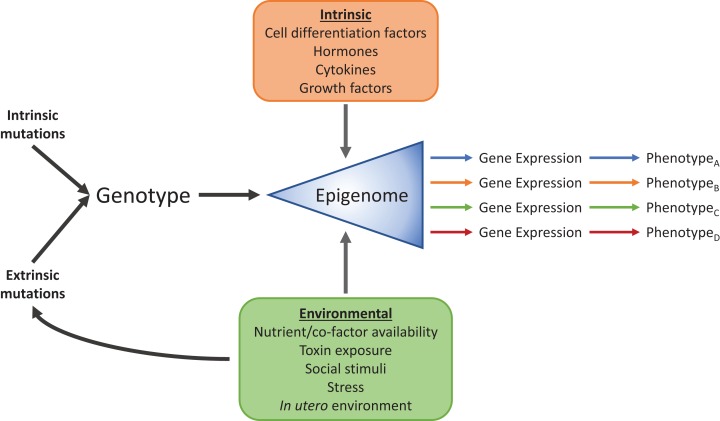
Intrinsic and environmental signals integrate through the epigenome to produce distinct phenotypes from identical genomic information. Standard cellular, biochemical, physiological, and developmental signals integrate with environmental factors such as diet, toxin exposure, stress, social interactions and developmental conditions through the epigenome to produce numerous phenotypes.

Research into the field of epigenetics has begun to reveal the mechanisms that allow a wide range of phenotypes to arise from identical genomic information. It has long been known that these phenotypic differences, between cell-types for example, are due to differential expression of subsets of genes from the common genomic pool and their interaction with the environment (Fig. 2), but how exactly each subset is chosen has remained elusive. Signals from the environment are programmed into the epigenetic “memory” to affect gene expression, and are necessary for processes such as cellular differentiation, development, and proper alignment of metabolic machinery to nutritional availability, among others. Epigenetic mechanisms, such as DNA methylation and histone modifications, add layers of regulatory information onto the DNA sequence. Genomic information is passed from generation to generation through DNA replication, and in a similar sense, these additional layers can transmit gene regulatory information mitotically, and in some cases, transgenerationally (Skinner, 2011). Due to the power, complexity, and sometimes fragility of the epigenome, we are becoming increasingly aware of its involvement in a substantial number of pathologies. For example, the epidemic increase in people with metabolic syndrome seen in the past several decades may, at least in part, be attributable to epigenomic misalignment between nutritionally deficient intrauterine conditions, and high-caloric diets later in life. Originally met with much skepticism, the “thrifty phenotype” hypothesis (Hales & Barker, 1992) postulated that maternal malnutrition programmed the developing fetus to be hypersensitive to macro- and micronutrients present in what was expected to be a nutrient sparse environment. When this anticipatory adaptation met a modern high-carbohydrate, high-fat diet, the result was increased susceptibility to T2D, obesity, and other hallmarks of metabolic syndrome. Originally supported by epidemiological evidence (Hales & Barker, 2001), the development of new molecular biology techniques and epigenetic discoveries have shown direct evidence for the “thrifty phenotype”, where epigenetic mechanisms link early life experience to glucose-insulin dysregulation (Park et al., 2008; Raychaudhuri et al., 2008; Heijmans et al., 2008; Ding et al., 2012). This “thrifty phenotype” is part of a broader study of the developmental origins of health and disease (DOHaD) that has spawned from epidemiological studies connecting normal and abnormal environmental conditions at a broad range on developmental stages to the development of disease (Wadhwa et al., 2009). Below we will explore examples from the DOHaD approach, such as the “thrifty phenotype,” where perturbations, genomically and through the environment, are translated into epigenetic dysregulation of metabolism, leading to disease.

### DNA methylation and histone modification

Nucleosomes consist of 146 base pairs of DNA wrapped around an octamer complex of histone proteins. These functional units of chromatin are not only needed for higher order compaction of DNA during cell division, but are also essential for controlling gene expression. Both major components of the nucleosome, DNA and histone proteins, can be chemically modified by enzymes as part of a reader-writer-eraser paradigm. Although the proteins that add, remove, and interpret the modifications are generally distinct from each other, they form complex interaction, and regulatory networks. What emerges is a propagative epigenetic program that recruits chromatin remodeling, gene regulatory, and transcriptional machinery to dynamically regulate transcription in response to intra- and extracellular stimuli.

DNA is methylated at the C^5^ position of cytosines, forming 5-methylcytosine (5mC), and occurs mostly at CpG dinucleotides (Arand et al., 2012). DNA methylation occurs in two contexts: the creation of new information via *de novo* methylation by the DNA methyltransferase enzymes DNMT3A and DNMT3B (Okano et al., 1999), and the duplication of that information during subsequent DNA replication by DNMT1. Once a methylation pattern is established by *de novo* methyltransferases, the Watson–Crick base pairing symmetry of the CpG dinucleotide allows DNMT1 to acts as a maintenance methyltransferase, where it copies the previous methylation pattern onto the newly synthesized strand during DNA replication (Robert et al., 2002). The inhibitory functions of DNA methylation on gene transcription are mainly mediated by methyl-CpG-binding domain containing proteins which recruit transcriptional corepressors, but there is evidence that methylated DNA can also sterically hinder some transcription factor binding (Watt & Molloy, 1988). DNA methylation is classically associated with gene silencing, however, the function of 5mC may be more nuanced than once thought depending on its position relative to gene regulatory elements and high density CpG regions (CpG islands) (Jones, 2012; Schübeler, 2015).

Histone proteins can also be post-translationally modified in a variety of ways. Histones have highly conserved N-terminal tails that protrude from the core of the nucleosome and when modified can either regulate the structure of chromatin directly, or serve to recruit effector proteins such as chromatin remodelers and transcription factors (for review see Martin & Zhang, 2005; Bannister & Kouzarides, 2011; Holt, Wang & Young, 2017). Although there are numerous histone modifications known (Kouzarides, 2007), and more being discovered each year (Tan et al., 2011; Chen, Miao & Xu, 2017), the best-characterized modifications are histone lysine acetylation and methylation. Lysine residues on histones are acetylated by histone acetyltransferases (HATs) and deacetylated by histone deacetylases (HDACs). The effect of histone acetylation is twofold: (1) it creates binding sites for factors involved in transcriptional regulation, typically in the context of activation, and (2) addition of an acetyl-group neutralizes the positive charge of the histone protein, weakening the histone-DNA interaction, relaxing chromatin conformation, and ultimately increasing DNA accessibility (Workman & Kingston, 1998; Bannister & Kouzarides, 2011). This second point contrasts histone lysine methylation which acts more classically as only an epigenetic “mark,” where it’s function is to be recognized by effector proteins that act positively or negatively on transcription (Martin & Zhang, 2005). Histone methylation also differs from acetylation in that up to three methyl groups can be transferred onto each lysine residue, each with potentially distinct functions. For example, histone H3 lysine 9 trimethylation (H3K9me3) is associated with transcription start sites of silent genes, whereas monomethylation (H3K9me1) is found in mostly active gene promoters (Barski et al., 2007).

**Figure 3:**
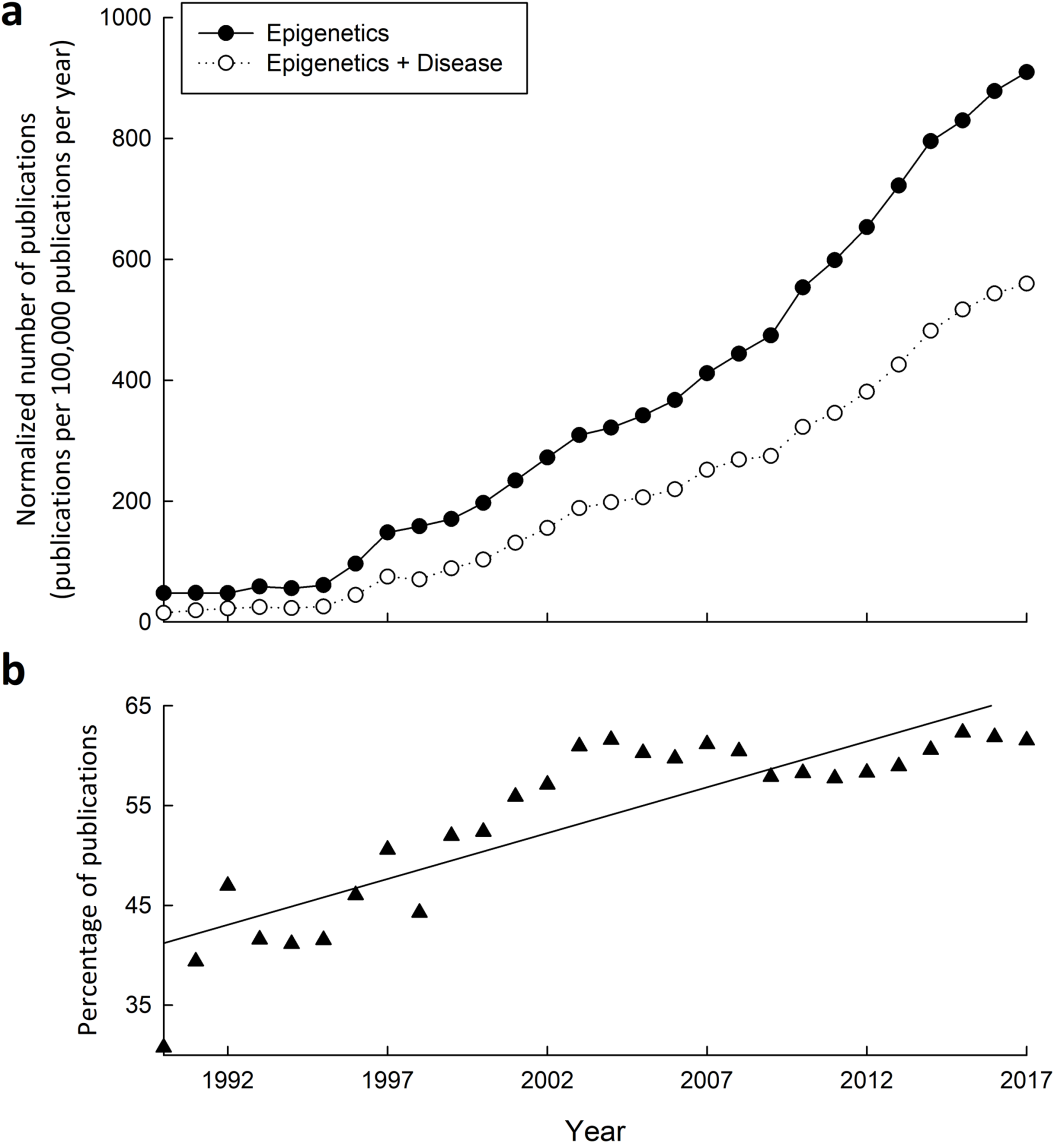
Research into the connections between epigenetics and disease has greatly increased from 1990–2015. (A) Yearly number of publications on PubMed matching combinatorial search results of “epigenetics” keywords alone, or paired with “disease” keywords. The following search queries were used for “epigenetics”: (epigenetic OR epigenetics OR epigenome OR “histone modification” OR “DNA methylation” OR “histone methylation” OR “histone acetylation”), and “epigenetics and disease”: (epigenetic OR epigenetics OR epigenome OR “histone modification” OR “DNA methylation” OR “histone methylation” OR “histone acetylation”) AND (disease OR disorder OR cancer OR syndrome). (B) Yearly percentage of epigenetics articles (from A) that involve disease.

The intertwining connections between epigenetic mechanisms and disease is clear given the power they hold over transcription. Great interest into these connections is exemplified not only by the fact that publications on epigenetics have increased significantly in the past two decades (Fig. 3A), but also by the increasing proportion of publications relating epigenetics to disease (Fig. 3B). Diseases attributable to epigenetic misregulation fit into two categories: (1) epigenetic dysfunction caused by mutations or otherwise improper function of epigenetic-related machinery, or (2) properly functioning epigenetic mechanisms producing inappropriate programming, inherited transgenerationally or programmed from previous life events. While common genetic and proteomic tools can help determine and rationalize the effects that a mutation may have, it is proving much more difficult to probe the epigenetic component of disease when epigenetic mechanisms are otherwise functioning normally.

### Epigenetics of diabetes and metabolic syndrome

A growing body of research using the DOHaD approach is showing that exposure to improper nutrition or endocrine disruption in utero produces epigenetic marks that are carried through life. A rat model of intrauterine growth retardation (IUGR) is used to study the effects of poor placental nutrient transport during pregnancy and has previously been associated with development of T2D in offspring (Simmons, Templeton & Gertz, 2001). In this model, *Pdx1*, a transcription factor that regulates β cell differentiation, and whose disruption causes T2D (Hani et al., 1999; Macfarlane et al., 1999; Leibowitz et al., 2001), was shown to have increased promoter DNA methylation, histone H3 and H4 deacetylation, and H3K9 dimethylation in IUGR pups (Park et al., 2008). Each of these epigenetic changes are associated with gene silencing, and in this case, were accompanied by inhibited *Pdx1* promoter binding by the transcription factor USF-1, and a 50% decrease in *Pdx1* mRNA levels in fetuses and pups that persisted into adulthood. *Pdx1* expression was then rescued by a short treatment with a HAT activator in newborn pups (Park et al., 2008; Pinney et al., 2011). These results demonstrate that not only can an aberrant transcriptional program be set early in life, but intervention and reversal is possible.

The transcriptional coactivator PGC-1α also has altered epigenetic regulation in T2D. The gene for PGC-1α, *PPARGC1A*, was shown to have increased promoter DNA methylation in pancreatic islet cells of type-2 diabetics compared to non-diabetics. This increase in DNA methylation was correlated with decreases in *PPARGC1A* expression and insulin secretion (Ling et al., 2008). PGC-1α is involved in regulating glucose homeostasis by co-activating FOXO1 (Puigserver et al., 2003), and polymorphisms in *PPARGC1A* are associated with increased risk for T2D (Pihlajamäki et al., 2005; Yang et al., 2011), which may play a similar functional role in modifying PGC-1α activity as promoter DNA methylation.

The histone H3 lysine 9 demethylase KDM3A (also known as JHDM2A) is crucially involved in genes that regulate energy and fat metabolism. *KDM3A^−/−^* mice have many of the signs of metabolic syndrome including insulin resistance, hypertriglyceridemia, elevated leptin levels, and obesity (Inagaki et al., 2009). Eliminating *KDM3A* resulted in the expected increase in gene-silencing H3K9me2 levels, and indeed several genes related to anti-adipogenesis, obesity and T2D were downregulated in the knockout mice. In a corollary study, instead of artificially increasing H3K9 levels by knocking out *KDM3A*, obesity was induced dietetically in dams, and then the effect on H3K9 levels in offspring was examined (Masuyama & Hiramatsu, 2012). Not only did these offspring perform worse on glucose tolerance tests, and show increased insulin resistance, but levels of leptin and adiponectin were significantly increased and decreased, respectively, which are both changes associated with obesity (Ukkola & Santaniemi, 2002; Enriori et al., 2006; Sáinz et al., 2015). Increased levels of H3K9me2 were found in the promoter of adiponectin, whereas increased levels of H4K20me1 (a permissive histone modification) was found in the leptin promoter, suggesting that histone modifications play a significant role in conferring obesity and metabolic syndrome phenotypes.

The effect of unfavorable environmental conditions early in life in humans is exemplified by extensive literature on prenatal exposure to famine. The best case study is that of the Dutch famine during the winter of 1944–1945, where those exposed prenatally showed phenotypes such as decreased glucose-tolerance (Ravelli et al., 1998), obesity (Ravelli, Stein & Susser, 1976; Ravelli et al., 1999), and even increased brain abnormalities and mental disorders (Susser et al., 1996; Susser, Hoek & Brown, 1998; Brown et al., 2000; Hulshoff Pol et al., 2000). The exact mechanisms at play are not completely understood, but mounting evidence suggests that altered epigenetic regulation may be responsible. Using exposed-unexposed same-sex sibling pairs, it has been shown that genes highly involved in metabolism had significantly altered methylation levels in the famine exposed-sibling (Heijmans et al., 2008; Tobi et al., 2009). *IGF2* hypomethylation was seen in males exposed to famine prenatally, a result that has also been correlated between obese fathers and their offspring (Soubry et al., 2013). IGF2 is an important growth factor during and after gestation, and therefore variable DNA methylation levels at the *IGF2* gene could influence *IGF2* expression and subsequent growth rate. Indeed, this hypothesis has some support in that brain weight is correlated with *IGF2* methylation in males (Pidsley, Dempster & Mill, 2010). Brain weight is decreased in schizophrenia, which raises an interesting suggestion that increases in schizophrenia associated with prenatal famine exposure may be mediated by changes in DNA methylation at the *IGF2* locus, although this will involve much more research. More recently, genome-wide approaches to DNA methylation have explored the epigenomic effects of prenatal famine (Tobi et al., 2014). Of interest is the finding that the insulin receptor gene (*INSR*) is hypermethylated in exposed individuals, and the levels of DNA methylation at the *INSR* gene is positively associated with both birth weight and LDL cholesterol. This group also summarized differentially methylated GO terms with REVIGO analysis and found that pathways involved in metabolism of triglycerides and lipids, regulation of growth, polysaccharide biosynthesis, and response to activity were among those significantly affected by prenatal famine exposure.

These results are beginning to show how adverse early-life conditions are predisposing affected individuals to disease in late-life, however, as with other epidemiological studies, this research area faces challenges in determining causality (Richmond et al., 2014). The infeasibility of randomized control trials means that confounding factors, selection bias, measurement and exposure inconsistencies between studies, and reverse causation need to be accounted for when taking a DOHaD approach (Gage, Munafò & Davey Smith, 2016; Yamada & Chong, 2017). The primary mechanism that is thought to translate changes in the epigenome to a specific phenotype is the influence of epigenetic marks on transcription factors. Transcription factor binding is influenced by the local epigenetic landscape, either directly through changes to the binding interface, or indirectly by influencing the recruitment of other transcriptional machinery. Examples of DOHaD studies directly addressing this mechanism are sparse (Martin & Fry, 2016; Tilley et al., 2018), and most epigenome wide association studies focus on enrichment of the epigenetic marks themselves. Future studies should evaluate not only changes to the epigenome, but also the mediators of their effects.

### Gestational diabetes and the epigenome

Gestational diabetes mellitus (GDM) is glucose intolerance arising during pregnancy in non-diabetic women. GDM creates a hyperglycemic intrauterine environment, which predisposes offspring to a variety of metabolic disorders later in life such as T2D (Clausen et al., 2008; Dabelea et al., 2008), metabolic syndrome (Boney et al., 2005; Clausen et al., 2009), and possibly childhood obesity (Gillman et al., 2003; Kim et al., 2011). GDM constitutes a form of *in utero* over-nutrition, and yet offspring have similar outcomes to those who experience under-nutrition, which has led to the observation of a U-shaped relationship between metrics such as birthweight and T2D or obesity (Pettitt & Jovanovic, 2001; Harder et al., 2007). These results suggest that GDM offspring have altered developmental programming, which has led to several studies examining the epigenetic impact of this *in utero* environment. A genome-wide DNA methylation analysis of control and GDM offspring revealed 1,373 and 1,418 methylation variable positions (MVPs) in placenta and cord blood, respectively (Finer et al., 2015). These results displayed tissue-specific differences in MVP enrichment relative to genomic features and CpG islands, suggesting a nuanced, rather than general, effect on DNA methylation patterns. KEGG analysis revealed a significant enrichment of MVPs in pathways such as endocytosis, chemokine signaling, and those related to cancer and metabolism, suggesting altered gene regulation, and potential mechanisms conferring metabolic pathogenesis later in life. These interpretations agree with a smaller study that examined changes in DNA methylation genome-wide and found that indeed genes associated with metabolic diseases were affected in infants exposed to GDM (Ruchat et al., 2013).

Other studies have taken a narrower approach to studying the consequences of GDM, focusing on specific genes. El Hajj et al. (2013) found that the imprinted *MEST* gene had lower DNA methylation levels in offspring of both dietetically- and insulin-treated GDM mothers. This gene has not only been found to be upregulated in obese humans (Kosaki et al., 2000) and mice (Takahashi, Kamei & Ezaki, 2005), but is correlated specifically with fat-mass expansion (Koza et al., 2006; Jura et al., 2016). The authors suggest that the decrease in *MEST* DNA methylation may contribute to the risk of developing obesity by these offspring later in life (El Hajj et al., 2013). Another group found increased leptin gene (*LEP*) promoter methylation in GDM offspring using results from the Rhode Island Child Health Study (Lesseur et al., 2014b). Leptin is the adipokine that signals satiety when energy stores are high, and leptin resistance is a hallmark of obesity, meaning satiety signaling is disrupted compared to non-obese subjects (Enriori et al., 2006). Interestingly, this same group found that *LEP* promoter DNA methylation was positively associated with lethargic behavior in male infants (Lesseur et al., 2014a). Indeed, increased *LEP* promoter DNA methylation occurs in rats with diet-induced obesity as well (Milagro et al., 2009), which opens the possibility that the increased methylation seen in GDM offspring may interact with their susceptibility to obesity. In contrast to promoter sites, another group suggested a causal link between maternal hyperglycemia and hypomethylation of a CpG site within the first intron of *LEP*, which was negatively correlated with cord-blood leptin levels (Allard et al., 2015). The authors suggest that given the causal role between GDM and epigenetic regulation of leptin, early intervention by epigenetic modification may mitigate the negative metabolic effects seen later in life.

Studies exploring the epigenetic link between GDM exposure and disease are limited primarily by their correlative nature. Determining cause-and-effect would require interventions that are generally not possible technologically or ethically, thus our understanding of these processes is currently bounded to associative studies. For obvious reasons, studies involving epigenetic analysis of neonates are confined to the placenta and cord blood. This limits the extrapolation power of this research given how tissue-specific epigenetic programming can be. This area will benefit from longitudinal studies linking the epigenome of perinatal organs to the epigenome of organs and tissues associated with the development of disease later in life.

### Methyl-donor availability and methyl-metabolism

Many metabolites serve as cofactors or substrates for epigenetic enzymes (Katada, Imhof & Sassone-Corsi, 2012; Keating & El-Osta, 2015). Of particular importance is S-adenosyl methionine (SAM), which is the sole methyl-donor for both DNA and histone methylation. The transfer of the methyl-group from SAM by DNA or histone methyltransferases results in the formation of S-adenosyl homocysteine (SAH), which in turn inhibits the activity of those methyltransferases (James et al., 2002). This means that modulation of SAM, SAH, and other one-carbon pathway metabolite levels can passively regulate the activity of epigenetic methyltransferases, directly linking the epigenome to metabolic status. In fact, it’s been shown that disrupted methyl-group metabolism in transgenic mice leads to general hypomethylation of DNA (Chen et al., 2001; Poirier et al., 2001). SAM is mostly derived from dietary methyl-donors such as folate, choline, and methionine, which indicates that methylation patterns, and therefore transcriptional patterns, can be altered directly by dietary insufficiencies (Wainfan et al., 1989; Tsujiuchi et al., 1999). Methyl-deficiency and subsequent general DNA hypomethylation has long been known to be carcinogenic (Wilson, Shivapurkar & Poirier, 1984; Lin et al., 2001; Gaudet et al., 2003), specifically hypomethylation of oncogenes creates a transcriptional environment in which they can be expressed (Feinberg & Vogelstein, 1983; Bhave, Wilson & Poirier, 1988; Zapisek et al., 1992; Wu et al., 2005).

A powerful tool for studying the epigenetic consequences of dietary and environmental factors is the viable yellow agouti (A^vy^) mouse model. In these mice, the upstream insertion of an intracisternal A particle (IAP) retrotransposon causes constitutive expression of the *Agouti* gene, which is normally silenced by DNA methylation (Dolinoy, 2008). Due to global epigenetic reprogramming events early in development (for review see Cantone & Fisher, 2013), disruption in the DNA methylation pathway at this time can leave this IAP unmethylated. Offspring with full methylation capabilities are brown, whereas those with reduced DNA methylation, either through dietary deficiencies or non-functional epigenetic machinery, have more yellow in their coats as DNA methylation levels decrease (Dolinoy, 2008). This means that the availability of methyl-donors and proper methyl-metabolism in utero not only has transcriptional consequence, but the effect of environmental stimuli on DNA methylation can be measured by proxy through coat color. This system has been used to study the effects of methyl-donor supplementation during pregnancy, such as folate, which is recommended to prevent neural tube defects. Researchers have found that maternal methyl supplementation indeed increases global DNA methylation in offspring (Cooney, Dave & Wolff, 2002; Waterland & Jirtle, 2003), and may modify the germline epigenome and therefore extend to further generations (Cropley et al., 2006). Methyl-donor supplementation has since been shown to counteract potentially pathogenic DNA hypomethylation caused by maternal exposure to the endocrine disruptor bisphenol A (BPA) (Dolinoy, Huang & Jirtle, 2007). In this study, Dolinoy, Huang & Jirtle (2007) found a >30% decrease in average methylation of nine CpG sites in the A^vy^ IAP in response to supranormal BPA treatment, and a similar decrease in the *Cabp^IAP^* metastable epiallele, suggesting high levels of BPA affects DNA methylation globally, rather than at specific loci. BPA exposed mice supplemented with a mixture of methyl-donors (folic acid, vitamin B_12_, betaine, and choline chloride) had restored DNA methylation levels and reduced yellow coat color compared to mice without supplementation. Similar results have been shown in human tissues with detectable levels of BPA (Faulk et al., 2016); however, the efficacy of methyl-donor supplementation for reducing the harmful effects of prenatal BPA exposure in humans in still an open question.

### Cancer metabolism and epigenetics

The field of cancer epigenomics is vast and extensively reviewed (Esteller, 2008; Sharma, Kelly & Jones, 2010; Rodríguez-Paredes & Esteller, 2011; Dawson & Kouzarides, 2012; You & Jones, 2012) which is why for this review we will stay within the context of metabolism and epigenetics with respect to cancer. Cancer is a broad grouping of diseases with an extremely large range of phenotypes and clinical implications, all with the commonality of unrestricted cell proliferation. Besides bypassing checkpoint mechanisms that regulate normal cellular function, cancer cells often have modified metabolic profiles that support continued growth and cell division.

Recently, the term “oncometabolite” has been coined to describe metabolites associated with cancer and oncogenesis. These are normally produced metabolites, but gain “onco”-status through mutations and malfunctions of metabolic enzymes leading to their abnormal accumulation found in cancer cells. Metabolic enzyme mutations are common in various cancers, for example, mutations in succinate dehydrogenase (SDH) are present in paragangliomas, gastrointestinal stromal tumors, neuroblastomas, renal tumors, and thyroid carcinomas (Bardella, Pollard & Tomlinson, 2011), isocitrate dehydrogenase (IDH) mutations are in many central nervous system cancers, leukemia, prostate cancer, and colon cancer (Yen et al., 2010), and fumarate hydratase (FH) mutations are found in fibroids, skin, and renal cancers (Tomlinson et al., 2002; Toro et al., 2003) among others. Oncometabolites are proving to play many roles in cancer but of particular interest here is their action on the epigenome (Yang, Soga & Pollard, 2013). Mutations in SDH, IDH, and FH lead to accumulation of succinate, D-2-hydroxyglutarate (2HG) and fumarate, respectively, which then have competitive inhibitory effects on DNA and histone demethylase enzymes (Xiao et al., 2012), suggesting that elevated methylation will result. SDH mutations in paragangliomas show hypermethylation in gene promoters, particularly associated with genes involved in neuroendocrine differentiation, leading to their downregulation (Letouzé et al., 2013). Inhibiting SDH pharmacologically increases multiple histone H3 methylation marks which can then be rescued by the overexpression of the histone demethylase JMJD3 (Cervera et al., 2009). SDH can also be silenced epigenetically, seen in a patient with paragangliomas, which could provide a positive feedback cycle where epigenetic silencing of SDH causes epigenetic silencing of other genes (Richter et al., 2016). FH mutants exhibit similar phenotypes to SDH mutants given their close metabolic relationship; both have shown to reduce 5hmC, which is one of the first products in the TET2-dependent DNA demethylation pathway (Xiao et al., 2012). In the context of oncometabolomics, SDH and FH mutants are loss-of-function mutations whereas IDH1/IDH2 mutants are gain-of-function, producing excess 2HG, however the resultant inhibition of demethylation is similar. Multiple studies have shown that IDH1/IDH2 mutations present in leukemia cause the accumulation of 2HG, inhibiting DNA demethylation and producing a hypermethylation phenotype. Hypermethylation in hematopoietic stem cells suppresses genes needed for differentiation, causing an increase in progenitor cells and a proleukemogenic effect (Figueroa et al., 2010; Sasaki et al., 2012). IDH1/2 mutations are also present in approximately 80% of grade II-III gliomas (Cohen, Holmen & Colman, 2013), producing a CpG island hypermethylator phenotype (Noushmehr et al., 2010). This phenotype was replicated in immortalized primary human astrocytes containing the predominant IDH1 mutation (R132H) found in low-grade gliomas (Turcan et al., 2012), demonstrating the causal role of this single mutation. The L-2HG enantiomer also presents oncometabolic DNA demethylation inhibitory effects in kidney tumors, however this is due to changes in L-2HG dehydrogenase rather than IDH1/IDH2 (Shim et al., 2014).

One of the most common metabolic shifts in cancer cells is the production of lactate from glucose even when oxygen is present, known as the Warburg effect (Kim & Dang, 2006). While the mechanisms and utility of the Warburg effect are still unclear (Liberti & Locasale, 2016), modern epigenetic contributions have been added to the debate in an area that was dominated by classic genetic and biochemical analysis (Wang & Jin, 2010). In gastric cancer cell lines, fructose-1,6-bisphosphatase (FBP) is downregulated in an NFκB-dependent manner with Ras-mediated transformations (Liu et al., 2010). When NFκB is inhibited in these cells, the *FBP1* promoter is demethylated and expression of FBP increases, decreasing cell proliferation. This same study found that the methylation status of the *FBP1* promoter alone can predict prognosis of gastric cancers, where individuals with unmethylated promoters have higher survival rates.

Of course, the broader metabolic status can also predispose an individual to developing cancer. As previously mentioned, simple dietary insufficiencies of metabolites necessary for proper methylation can result in global DNA demethylation, potentially unleashing oncogenes. Reversing this situation by increasing one-carbon metabolite supplementation may be a clinically relevant intervention as it has been suggested to decrease multiple cancers including breast (Xu et al., 2009), colorectal (Kim, 2007), and lung (Heimburger et al., 1988), however few studies have explored whether this is an epigenetic mediated effect (Kim et al., 2001a; Du et al., 2009).

These are just a few examples that show the interconnectedness of metabolism, epigenetics, and cancer. Much research is needed to determine exactly how much each genetic, epigenetic, and environmental factor contributes to oncogenesis. The result of these studies will allow for the development of pharmacological or targeted epigenetic interventions to compliment or replace the current portfolio of chemotherapeutic and radiological treatments.

## Control of Metabolism: Lessons from Nature

Natural models for manipulating metabolism can serve as great resources for inspiring treatments for many diseases. Our lab and others study animals that can survive freezing, anoxia, severe dehydration, and hibernation. Animals that survive these conditions all use metabolic rate depression and metabolic reorganization as adaptations to conserve energy and enact cytoprotective strategies. For example, the wood frog (*Rana sylvatica*) survives whole body freezing in the winter, in part by producing huge amounts of glucose as a cryoprotectant, elevating plasma glucose levels to 200 mM (human type-2 diabetics >10 mM are considered hyperglycemic), and yet do not suffer the damage or cytotoxicity associated with diabetes (Storey & Storey, 1984). Much research has been done to uncover the metabolic processes that allow this adaptation to occur (Storey, 1990; Storey & Storey, 2004, 2017), and we are just beginning to explore the potential involvement of epigenetic mechanisms. Our recent work has shown that the wood frog globally reduces H3K4me1 levels, a permissive histone modification, but maintains H3K9me3 content, a modification associated with gene silencing, while frozen (Hawkins & Storey, 2018). This result is in line with the metabolic reorganization that the wood frog undergoes where expression of most genes are downregulated to conserve energy resources under the anoxic conditions of the frozen state. We have also shown that frozen wood frogs suppress cyclins and cyclin-dependent kinases to gain potential energy savings by halting cell cycle activity (Zhang & Storey, 2012). Future studies will evaluate the involvement of other epigenetic mechanisms and their regulation of specific genes to further elucidate the mechanisms involved in surviving freezing and its associated extreme glucose levels.

Mammals such as the hibernating 13-lined ground squirrel (*Ictidomys tridecemlineatus*) may aid us in the quest to improve tissue and organ preservation for medical transplantation. When torpid they substantially decrease their body temperature and rates of heartbeat, breathing, and organ perfusion rate (Carey, Andrews & Martin, 2003; Storey & Storey, 2005). How they coordinate and regulate all of these may provide lessons which could help us extend organ storage time to increase the window of viability for successful transplantation. We have shown that during hibernation these animals increase protein and activity levels of HDACs and DNMTs, as well as global DNA methylation in a tissue specific manner (Morin & Storey, 2006; Biggar & Storey, 2014; Alvarado et al., 2015), all of which suggest global gene silencing is taking place. Moreover, differential regulation of microRNAs occurs in ground squirrels during hibernation and is postulated to be involved in regulating suppression of various cellular processes including reducing cell cycle activity during hibernation (Wu et al., 2016). Increased DNA methylation was also seen in anoxia tolerant turtles (*Trachemys scripta elegans*) (Wijenayake & Storey, 2016) that can survive without oxygen for weeks at a time. However, despite global metabolic rate depression, selective upregulation of some beneficial pathways is possible. For example, the DNA binding activity of NFκB increased in anoxic turtles, causing an increase in the transcription of downstream genes under its control (Krivoruchko & Storey, 2010). Other animals such as the naked mole-rat (*Heterocephalus glaber*) have yet to be studied in terms of epigenetic mechanisms, but they may potentially have much to offer towards our understanding of cancer, aging, and metabolic regulation since these animals are the longest-living rodents and are uniquely resistant to cancer (Buffenstein, 2005; Liang et al., 2010). Inspiration from these animals and others that can endure extreme environmental stress could be combined to create targeted therapeutics as treatments for disease.

## Conclusion

Regulation of gene transcription is multifaceted, with innumerable possibilities for errors to occur. Since metabolism both affects and is affected by transcriptional regulation, when errors do occur, metabolism at all scales is disrupted, from systemic insulin dysregulation in T2D to aerobic glycolysis in individual cancer cells. Each of the areas discussed in this review are still being actively researched and adding to our understanding of how we may be able to control transcription to treat these pathologies as they appear. The introduction of new technologies will further our abilities to probe the structure of genes, their functional elements, epigenetic regulation, and the interactions that each of these have with the environment.

## Supplemental Information

10.7717/peerj.5062/supp-1 Supplemental Information 1 Raw data for figure 3.

## Additional Information and Declarations

### Data Availability

The following information was supplied regarding data availability: The raw data are provided in a Supplemental File.
